# High-Pressure Structures and Superconductivity of Barium Iodide

**DOI:** 10.3390/ma15020522

**Published:** 2022-01-10

**Authors:** Shubo Wei, Hanyu Liu

**Affiliations:** 1 State Key Laboratory for Superhard Materials, College of Physics, Jilin University, Changchun 130012, China; weishubo.good@163.com; 2 International Center for Computational Methods and Software, College of Physics, Jilin University, Changchun 130012, China

**Keywords:** high pressure, superconductivity, crystal structure prediction, phase transition

## Abstract

Generally, pressure is a useful tool to modify the behavior of physical properties of materials due to the change in distance between atoms or molecules in the lattice. Barium iodide (BaI_2_), as one of the simplest and most prototypical iodine compounds, has substantial high pressure investigation value. In this work, we explored the crystal structures of BaI_2_ at a wide pressure range of 0–200 GPa using a global structure search methodology. A thermodynamical structure with tetragonal *I*4/*mmm* symmetry of BaI_2_ was predicted to be stable at 17.1 GPa. Further electronic calculations indicated that *I*4/*mmm* BaI_2_ exhibits the metallic feature via an indirect band gap closure under moderate pressure. We also found that the superconductivity of BaI_2_ at 30 GPa is much lower than that of CsI at 180 GPa based on our electron–phonon coupling simulations. Our current simulations provide a step toward the further understanding of the high-pressure behavior of iodine compounds at extreme conditions.

## 1. Introduction

A large number of interesting properties of iodine compounds have been reported such as optical properties, conduction characteristics, catalytic performance, medical application and the hypervalence phenomenon [1,2,3,4,5,6,7,8,9,10]. Barium iodide (BaI_2_), as one of the simplest and most representative ionic iodine compounds, has attracted plentiful studies [11,12,13,14,15]. At ambient pressure, barium iodide (BaI_2_) shares a cotunnite-type structure (orthorhombic *Pnma*, *Z* = 4) [11] with many other dihalides AX_2_ (A = Ca, Ba, Pb, Sn) [16] Under high pressure, a new high-pressure phase of BaI_2_ with monoclinic *P*112_1_/*a* (*Z* = 8) symmetry between 12 and 15 GPa was reported in 1995 [13]. Curiously, this previous work identified the monoclinic *P*112_1_/*a* phase at a pressure range of 20–40 GPa [13]. Moreover, the latest theoretical work in 2015 reported that BaI_2_ maintains insulator characteristics at least up to 40 GPa [15]. The superconducting behavior of CsI has been reported by experimental and theoretical works [7,17,18], where the external pressure reached 180 GPa. BaI_2_, as one of the most representative iodine compounds, shares a similar prototype with CsI. According to the reported superconducting mechanism of CsI, the increasing number of electrons of Cs is responsible for superconductivity, and the Ba atom with more electrons is potentially superior to the Cs atom in the electron–phonon coupling. Thus, it is an interesting topic to investigate the conduction characteristics at lowering pressure, which implies much more application value. To the best of our knowledge, the highest pressure studied in previous work for BaI_2_ was lower than 40 GPa, and the whole high-pressure structural transition sequence up to 200 GPa is still far from being clear and well established. These structural uncertainties impede the in-depth understanding and further exploration of the electronic properties of BaI_2_ under compression.

Here, we present systematic structure searches for BaI_2_ at high pressures using our developed CALYPSO method [19,20,21,22]. Our results show that BaI_2_ adopts orthorhombic *Pnma* symmetry at 0 GPa, which is consistent with experimental work [11,13]. Furthermore, we identified one thermodynamically stable tetragonal *I*4/*mmm* phase for BaI_2_ at high pressure which is energetically superior to the previously reported post-cotunnite phase (*P*112_1_/*a*, *Z* = 8) [13]. Further electronic calculations showed that BaI_2_ becomes a metallic phase via indirect band gap closure at moderate compression. In addition, the simulations also suggest the superconductive feature of BaI_2_ at 30 GPa [7].

## 2. Computational Details

To obtain stable high-pressure structures of BaI_2_, we carried out a global structure search by our developed CALYPSO (Crystal structure AnaLYsis by Particle Swarm Optimization) method [19,20,21,22] within the first-principle electronic structure. This computational scheme was verified by many works [23,24,25,26,27]. We used Perdew–Burke–Ernzerhof generalized gradient approximation (PBE-GGA) [28] exchange correlation for the simulations, where the VASP (Vienna ab initio simulation package) code was also employed [29]. Furthermore, we used the Phonopy code to investigate the dynamic properties of the predicted structures [30]. The Device Studio [31] program provides a number of functions for performing visualization, modeling and simulation. We used Bader’s quantum theory of atoms in molecules (QTAIM) analysis [32] to investigate the charge properties of the predicted structures. Superconductivity was studied by density-functional perturbation theory [33,34], where the Quantum-ESPRESSO package [35,36] was used.

## 3. Results and Discussion

**Figure 1:**
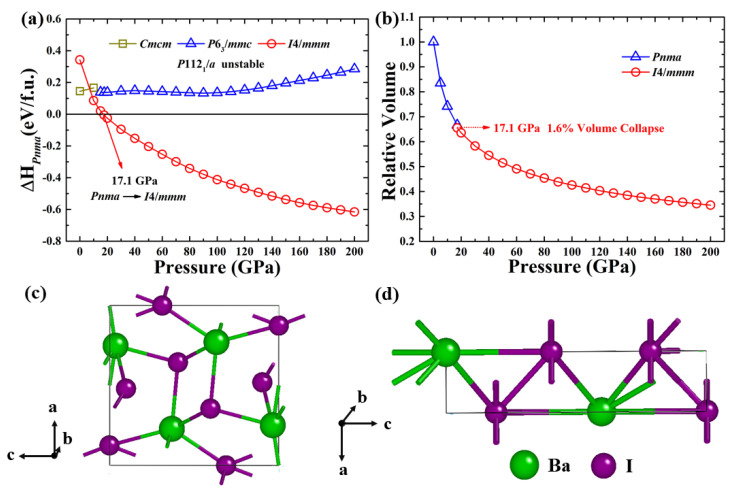
(**a**) Enthalpies (related to the *Pnma* phase) of *Cmcm*, *P6*_3_/*mmc* and *I*4/*mmm* structures of BaI_2_. The former reported *P*112_1_/*a* structure^13^ is thermodynamically unstable. (**b**) The relative volume of BaI_2_. The crystal structure of orthorhombic *Pnma* (**c**) and tetragonal *I*4/*mmm* (**d**) of BaCl_2_ at 0.3 and 17.1 GPa, respectively.

To uncover stable structures of BaI_2_, we performed systematic structural predictions at a pressure range of 0–200 GPa without considering temperature effects, with the CALYPSO methodology [19,20,21,22]. According to our simulations, at ambient pressure, the cotunnite-type *Pnma* (*Z* = 4) phase of BaI_2_ is the thermodynamically optimal structure (Figure 1). From Figure 1c, each barium atom was surrounded by nine iodine atoms, and all the atoms of the primitive cell were packed in an orthorhombic cage. The optimized lattice parameter of the *Pnma* phase takes *a* = 9.003 Å, *b* = 5.448 Å and *c* = 10.879 Å at ambient pressure, which is close to experiment data with *a* = 8.922 Å, *b* = 5.304 Å and *c* = 10.695 Å [11]. The agreement between our current simulations and experimental studies [11,13,15] provides a validation of the present simulation scheme.

**Figure 2:**
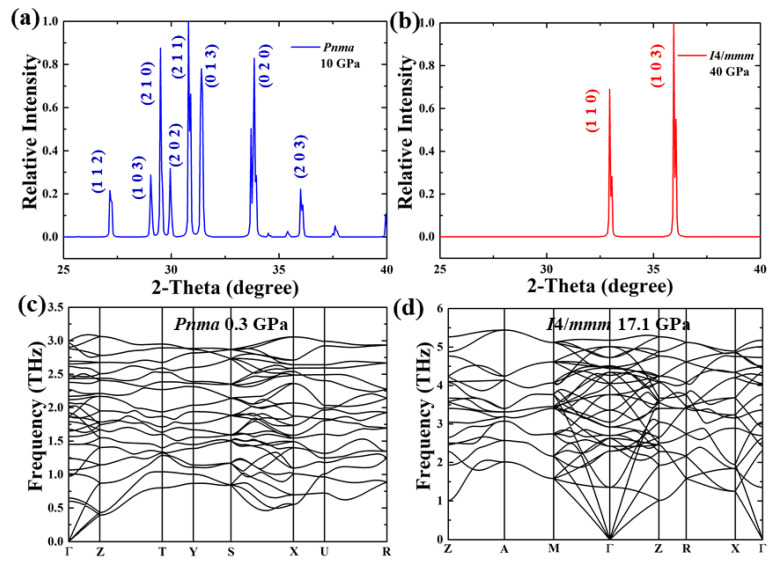
The simulated power X-ray diffraction, where λ of 1.54 Å was used for *Pnma* BaI_2_ at 10 GPa (**a**) and *I*4/*mmm* BaI_2_ at 40 GPa (**b**). Calculated phonon spectra of *Pnma* BaI_2_ at 0.3 GPa (**c**) and *I*4/*mmm* BaI_2_ at 75 GPa (**d**).

Under high pressure, we found the stability of the *Pnma* structure at a pressure range of 0–17 GPa, after which it undergoes a phase transition into the tetragonal *I*4/*mmm* phase (*Z* = 2), with nearly no volume collapse, as shown in Figure 1. Comparing with the *Pnma* structure, the *I*4/*mmm* phase has a higher symmetry with a coordination of 10. We provide the lattice parameters and atomic coordinates in Table 1. Earlier experimental work showed the existence of a monoclinic *P*112_1_/*a* phase of BaI_2_ at a pressure range of 12–15 GPa at room temperature.^13^ According to the lattice parameters and atomic positions in the current work, we calculated the enthalpy of the experimentally known monoclinic *P*112_1_/*a* structure, and our results indicate that this *P*112_1_/*a* structure has a higher enthalpy than our predicted structure. The enthalpy curve of *P*112_1_/*a* is not shown due to its large number compared with the reference enthalpy lines. The *P*6_3_*/mmc* and *Cmcm* phases, as underlying stable structures obtained during the structure prediction process, were also taken into account in the comparison (Figure 1a). We also provide the X-ray diffraction patterns of these structures in Figure 2, and the future experiment is thus stimulated. Our work shows that BaI_2_ follows the following structural progression: orthorhombic *Pnma* (0–17.1 GPa) → tetragonal *I*4/*mmm* (17.1–200 GPa). Our simulations also suggest the dynamical stability feature of the predicted structures, as shown in Figure 2.

**Figure 3:**
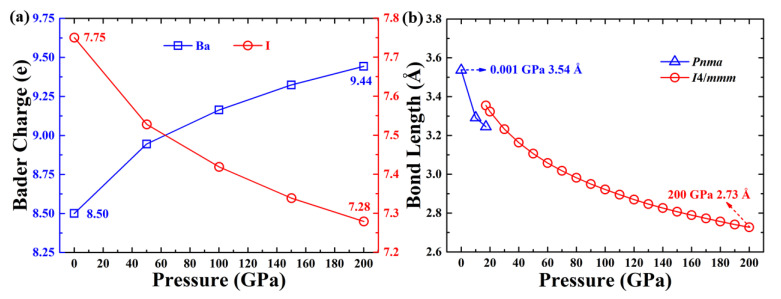
(**a**) Simulated Bader charge for BaI_2_. (**b**) The distance between B and I atoms.

In this work, we also provide the Bader charge and the Ba–I bond distance in Figure 3. These simulations suggest the peculiar reverse of the charge transfer between Ba and I atoms.

**Figure 4:**
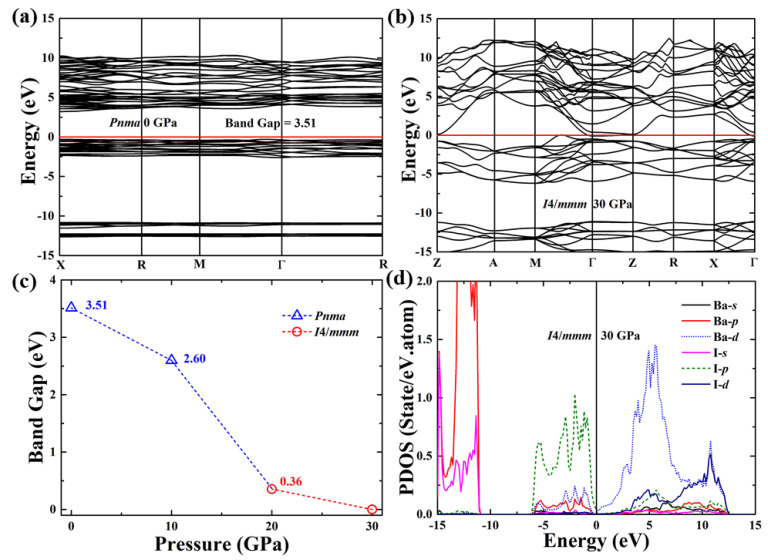
Calculated electronic band plot along high-symmetry directions of *Pnma* BaI_2_ at 0 GPa (**a**) and *I*4/*mmm* BaI_2_ at 30 GPa (**b**). Pressure dependence of theoretical band gaps of BaI_2_ (**c**). Electronic density of states of *I*4/*mmm* BaI_2_ at 30 GPa (**d**).

Previous studies suggested that CsCl, CsBr and CsI are metallic phases at high pressures [7,37]. At ambient conditions, BaI_2_ has a wide band gap between valance and conductive bands, with a value of 3.51 eV (Figure 4a). Interestingly, our studies also show the metallic feature of BaI_2_ at a moderate pressure of 30 GPa (Figure 4), which is quite different from the direct band gap closure in the CsCl and CsBr systems [37]. The latest theoretical work in 2015 reported that BaI_2_ maintains its insulating feature up to 40 GPa [15]. It is noteworthy that it adopts the ambient orthorhombic *Pnma* structure, which is obviously not accurate without considering high-pressure phases. Therefore, the newly predicted tetragonal phase with *I*4/*mmm* symmetry is responsible for this pressure-induced metallization.

**Figure 5:**
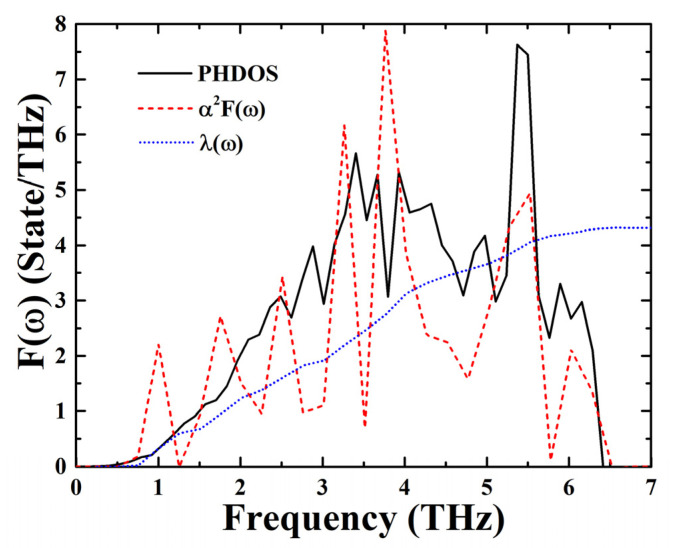
The electron–phonon coupling calculations of *I*4/*mmm* BaI_2_ at 30 GPa.

The superconducting behavior of CsI has been extensively explored by experimental and theoretical methods [7,17,18,19]. Therefore, it is noteworthy to study the superconductivity of BaI_2_ at high pressure. In this work, we performed electron–phonon coupling calculations for BaI_2_. We provide the Eliashberg spectral function *α^2^F*(*ω*) [38] (Figure 5) and the phonon density of states (PHDOS) of BaI_2_ at 30 GPa. It is clearly seen that *α^2^F*(*ω*) can be roughly divided into three parts: low (0.49–1.25 THz), medium (1.25–5.78 THz) and high frequency (5.78–6.51 THz). We employed the Allen–Dynes modified McMillan equation [39] $T_{c}=\frac{\omega_{\log\;}}{1.2}\exp\left[-\frac{1.04\left(1+\lambda \right)}{\lambda -\mu^{*}\left(1+0.62\lambda \right)}\right]$ to estimate the superconductivity of the predicted structures, where *ω*_log_ is the logarithmic average frequency, and μ* is the Coulomb pseudopotential. At 30 GPa, *ω*_log_ was 132.62 K, while at 200 GPa, it was 207.23 K. Furthermore, we found that the estimated superconducting critical temperature *T_C_* for BaI_2_ was 1.27 K at 30 GPa and 0.35 K at 200 GPa. Moreover, two recent studies reported non-adiabatic superconductivity in sibling BKBO [40] and low-dimensional LiC_6_ [41]. These studies presented a coherent interpretation of the superconductivity. In our work, the relatively lower bottom of the conduction band of BaI_2_ implies the potential influence of non-adiabatic effects, and this may be an interesting orientation in future work.

## 4. Conclusions

We investigated the high-pressure crystal structures and electronic properties of BaI_2_ up to 200 GPa. Our results suggest a thermodynamically stable tetragonal *I*4/*mmm* symmetry of BaI_2_ identified at 17.1 GPa. The systematic increase in the coordination number from 9 to 10 should be seen in many AX_2_ compounds. Furthermore, we uncovered that *I*4/*mmm* BaI_2_ becomes a metallic phase at moderate pressure, which is different to *Pbam* CsCl and *Pmma* CsBr (via direct band gap closure at the Γ point) [37]. Our simulations suggest the superconducting feature of the predicted structure for BaI_2_ at 30 GPa. Comparing with CsI, cesium xenide and barium xenide have a similar composition with more electrons, making them good subjects for study, with great potential to exhibit superconductivity at moderate pressure. Our work clarifies the high-pressure phase transition sequence of BaI_2_ up to 200 GPa, which represents a step toward the understanding of the behavior of AX_2_-type iodine compounds under extreme conditions.

## Figures and Tables

**Table 1:** Lattice parameters and atomic coordinates of BaI_2_.

Phase	Lattice Parameters (Å)	Atoms	*x*	*y*	*z*
*Pnma* BaI_2_	*a* = 9.003	Ba1 (4c)	0.24	0.75	0.61
0.001 GPa	*b* = 5.448	I1 (4c)	0.64	0.75	0.58
	*c* = 10.879	I2 (4c)	0.48	0.25	0.84
*I*4/*mmm* BaI_2_	*a* = 4.094	Ba (2a)	0.00	0.00	0.00
17.1 GPa	*b* = 4.094	I (4e)	0.50	0.50	0.16
	*c* = 10.434				
*I*4/*mmm* BaI_2_	*a* = 3.255	Ba (2a)	0.00	0.00	0.00
200 GPa	*b* = 3.255	I (4e)	0.50	0.50	0.17
	*c* = 8.695				

## Data Availability

The data presented in this study are available on request from the corresponding author.
